# Inter-individual consistency in habitat selection patterns and spatial range constraints of female little bustards during the non-breeding season

**DOI:** 10.1186/s12898-018-0205-9

**Published:** 2018-12-05

**Authors:** Francesc Cuscó, Laura Cardador, Gerard Bota, Manuel B. Morales, Santi Mañosa

**Affiliations:** 1 Departament de Biologia Evolutiva, Ecologia i Ciències Ambientals, Institut de Recerca de la Biodiversitat (IRBio), Universitat de Barcelona, Avinguda Diagonal 643, 08028 Barcelona, Spain; 2 Centre for Biodiversity and Environment Research, Department of Genetics, Evolution, and Environment, University College London, London, WC1E 6BT UK; 3 Biodiversity and Animal Conservation Lab, Forest Sciences Center of Catalonia (CTFC), Ctra. de St. Llorenç de Morunys Km 2, 25280 Solsona, Spain; 4 Terrestrial Ecology Group (TEG), Department of Ecology, Universidad Autónoma de Madrid, C/Darwin 2, 28049 Madrid, Spain

**Keywords:** *Tetrax tetrax*, Inter-individual habitat selection, Ranging behavior, Non-breeding season, Spatial eigenvector mapping

## Abstract

**Background:**

Identifying the factors that affect ranging behavior of animals is a central issue to ecology and an essential tool for designing effective conservation policies. This knowledge provides the information needed to predict the consequences of land-use change on species habitat use, especially in areas subject to major habitat transformations, such as agricultural landscapes. We evaluate inter-individual variation relative to environmental predictors and spatial constraints in limiting ranging behavior of female little bustards (*Tetrax tetrax*) in the non-breeding season. Our analyses were based on 11 females tracked with GPS during 5 years in northeastern Spain. We conducted deviance partitioning analyses based on different sets of generalized linear mixed models constructed with environmental variables and spatial filters obtained by eigenvector mapping, while controlling for temporal and inter-individual variation.

**Results:**

The occurrence probability of female little bustards in response to environmental variables and spatial filters within the non-breeding range exhibited inter-individual consistency. Pure spatial factors and joint spatial-habitat factors explained most of the variance in the models. Spatial predictors representing aggregation patterns at ~ 18 km and 3–5 km respectively had a high importance in female occurrence. However, pure habitat effects were also identified. Terrain slope, alfalfa, corn stubble and irrigated cereal stubble availability were the variables that most contributed to environmental models. Overall, models revealed a non-linear negative effect of slope and positive effects of intermediate values of alfalfa and corn stubble availability. High levels of cereal stubble in irrigated land and roads had also a positive effect on occurrence at the population level.

**Conclusions:**

Our results provide evidence that female little bustard ranging behavior was spatially constrained beyond environmental variables during the non-breeding season. This pattern may result from different not mutually exclusive processes, such as cost–benefit balances of animal movement, configurational heterogeneity of environment or from high site fidelity and conspecific attraction. Measures aimed at keeping alfalfa availability and habitat heterogeneity in open landscapes and flat terrains, in safe places close to breeding grounds, could contribute to protect little bustard populations during the non-breeding season.

**Electronic supplementary material:**

The online version of this article (10.1186/s12898-018-0205-9) contains supplementary material, which is available to authorized users.

## Background

Identifying the factors that affect ranging behavior of animals is a central issue to ecology and an essential tool for designing effective conservation policies [1, 2]. This knowledge provides the information needed to predict the consequences of land-use change on species habitat use, especially in regions subject to major habitat transformations, such as agricultural landscapes. However, this type of information is not always available, particularly in the non-breeding season or non-breeding areas of species with different seasonal distribution ranges [3, 4]. Locations of radio and satellite telemetry provide valuable information on animal movement and are widely used for studies of habitat selection [5–7]. However, this type of data is frequently spatially autocorrelated [8, 9]. Spatial autocorrelation in animal movement is often assumed to be the result of species-specific responses to spatially structured environment. However, other ecological processes, such as cost–benefit balances of animal movement across space and time, dispersal limitations or conspecific attraction, can also be involved in generating this pattern [10]. Thus, accounting for spatial autocorrelation in analyses is essential to understand the ecological processes underlying ranging patterns [5].

Among environmental factors, habitat characteristics and resource availability have been identified as major factors determining ranging behavior in birds, e.g. [7, 11]. Other environmental factors such as risk of predation or anthropogenic conditions can also be involved [12, 13]. Additionally, individuals within a population can show different strategies in the way they exploit available habitat or space [14–16]. Indeed, individual differences in behavior, morphology, physiology, personality and life history traits are common across taxa, which may lead to inter-individual differences in cost–benefit balances of different ranging strategies [17–19]. Behavioral variability in a population is thus an issue to take into account to develop effective conservation measures.

The little bustard (*Tetrax tetrax* L.) is a medium-sized steppe bird that inhabits natural steppes and cultivated areas of the Palearctic [20]. This is a ground nesting bird with a characteristic polygynic mating system based on exploded leks [21]. During the non-breeding season the species is gregarious, forming large mixed flocks up to hundreds of individuals [22–24], even a few thousands in some regions [25]. The species has been described as migrant in Russia, central Asia and northern France and as sedentary or partially migrant—with variations between and within populations—in the Iberian Peninsula, Italy and southern France [20, 26, 27]. The species is listed as “Near Threatened” at global scale [28] and “Vulnerable” in Europe [29]. The Iberian Peninsula holds one of the most important populations in the world [23]. There, the species is undergoing rapid declines regionally [30–32], leading to a global decrease up to 50% of the Spanish population in a decade [33]. Contributing factors to such decline around the world are the loss and degradation of habitat related with agricultural intensification and hunting pressure [34–36]. Although habitat requirements during the breeding season have been the subject of much research [11, 37–41], studies centered on the non-breeding period are scarce (but see [42–44]), even when the species spends around 3/4 of its annual cycle (from July to February) in non-breeding grounds [27]. In addition, most studies on the species are based on male observations, while female ecology and behavior have been scarcely studied. This is largely due to the extremely secretive behavior of females in spring and consequently the difficulty of obtaining data about them [20].

In this study, we evaluate the role of environmental predictors in limiting ranging behavior during the non-breeding season of female little bustards in an intensified farmland, while considering potential spatial constraints in ranging patterns. We did so by implementing habitat selection models constructed with orographic, crop types, human-related variables and by excluding or incorporating spatial constraint variables obtained by eigenvector mapping. The inclusion of spatial constraint variables in models has recently been shown to effectively capture the effect of subjacent spatial structures that are not related to the environmental factors considered in models [45–47]. We also assessed for inter-individual variation in the importance of response to ecological requirements and spatial constraints by incorporating a random structure in models. Finally, we use our results to propose some recommendations in order to design future conservation actions to reconcile agricultural development and other potentially conflicting human activities with the preservation of little bustard populations during the non-breeding season.

## Methods

### Study area

**Fig. 1:**
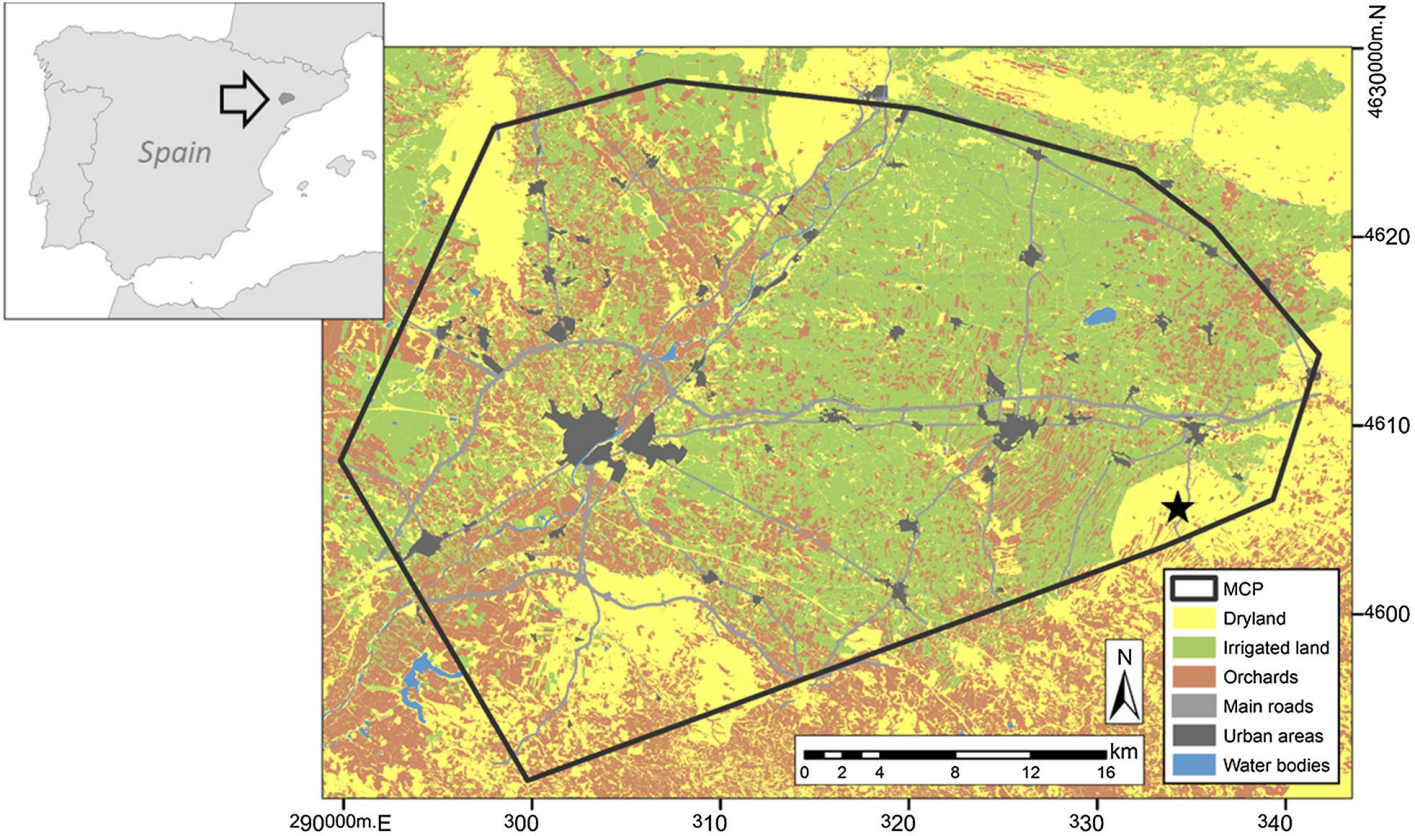
Location and land-use composition of the study area. Boundaries are defined by the minimum convex polygon (MCP) including the pool of locations of tagged female little bustards. Lleida is the large urban area located on the left. The black star indicates the main breeding area for the species in the study area. Reference coordinates in UTM. Map derived from SIGPAC cartography

The study area is located in the Plana de Lleida (UTM Y: 4592–4629 km N; X: 289–341 km E), a large agricultural area situated in the northeastern Ebro basin (Iberian Peninsula) at 261 ± 65 m over sea level. Climate is semiarid Mediterranean, with an average annual rainfall of 300–400 mm and an average temperature of 7–8 °C in winter and 24–25 °C in summer [48]. In the last decades, the study area has suffered a strong process of agricultural intensification with an increase in mechanization, the use of chemical fertilizers and pesticides, land concentration processes and the transformation of dry lands to irrigated crops [49]. Nowadays, the zone is dominated by intensively irrigated cultivated farmland, although extensive dry lands are present in the periphery of the area, where the main breeding grounds of the species in the region are located (Fig. 1). Drylands are dominated by winter cereals (mainly barley and wheat), as well as some scattered almond and olive tree groves. Irrigated crops include fruit orchards, corn, alfalfa and spring cereals. In autumn, with the beginning of a new agricultural cycle, most of cereal and corn fields are stubbles that will be plowed in the course of the season to turn into new sowings. Natural vegetation is scarce in the study area, representing around 10% of total surface.

### Telemetry data

From 2009 to 2013, 18 female little bustards were captured using adapted funnel traps (see [50]). The females were tagged with 22 g Solar Argos/GPS PTT-100 transmitters (Microwave Telemetry). The transmitter weight with harness never represented more than 5% of the body weight of the birds to avoid overload [9]. To avoid capture myopathy, the time of capture and handling were minimized and never exceeded 15–20 min [51, 52].

A maximum of six locations per transmitter and day were obtained for each female in non-breeding period (23th September–20th December) 2009–2013, with a latitude/longitude accuracy of 18 m (Microwave Telemetry). The study period was selected to ensure that all tracked birds had finished breeding or rearing chicks (the end of the breeding period and chick rearing period largely varies across individuals—from July to August—in the study area [49]) and to maximize data quality (the long periods of fog during winter in the study area did not allow the batteries recharge, leading to important information gaps). To homogenize the data from the different transmitters (oldest devices were programed to transmit only one location during non-breeding season to avoid the drain of batteries caused by long periods of fog), only one location per day for each female was used. For location selection we chose the common hour for all transmitters (14:00 GMT). In case this was not available, the nearest location in time was used. For 7 females complete data for the study period was not available due to mortality or device failure (n = 6), or abandonment of the study area (n = 1). These females were excluded from analyses. Thus, final data included 1841 locations for a total of 11 female little bustard for the whole study period. Final sample size per bird and season ranged from 34 to 89 locations (mean ± SD: 76.7 ± 16.1). Locations were incorporated into a Geographic Information System (GIS) using a Universal Transverse Mercator (UTM) grid of 1-km^2^ to fit environmental data resolution (see below). Duplicate samples (i.e. two or more records within the same grid cell) within same female and non-breeding season were handled as single observations, since we were interested in a presence/absence approximation of data. Association in flocks between tracked females was low (see Additional file 1) and thus data can be reasonably considered independent.

### Habitat variables

We compiled landscape composition data on five dominant crop cover type variables to represent habitat variability in the study area as well as human influence predictors and terrain slope (Table 1). These variables are likely to affect the species ranging patterns during the non-breeding period due to their different vegetation structure and management that affect provision of food availability, food accessibility and shelter [42–44, 53].

**Table 1:** Description of habitat variables (mean ± standard deviation), measured on each 1 × 1 km^2^, included in occurrence probability models of female little bustards in the Plana de Lleida (2009–2013)

Variable	Description	Source	Mean ± SD
Alfalfa	Proportion of alfalfa	SIGPAC and DUN	0.11 ± 0.10
Corn stb.	Proportion of corn stubble/plow	SIGPAC and DUN	0.15 ± 0.15
D. cereal stb.	Proportion of cereal stubble/plow in dryland	SIGPAC and DUN	0.09 ± 0.20
I. cereal stb.	Proportion of cereal stubble/plow in irrigated land	SIGPAC and DUN	0.11 ± 0.10
Orchards	Proportion of orchards	SIGPAC	0.24 ± 0.19
Roads	Proportion of roads	SIGPAC	0.01 ± 0.02
Terrain slope	Standard deviation of elevation (m)	DEM (15 × 15 m pixel)	6.58 ± 4.80
Urban areas	Proportion of urban areas	SIGPAC	0.03 ± 0.11

The habitat variables were acquired combining the information from Geographic Information System of Farming Land (SIGPAC versions 2009–2013)—which provides information on large groups of land uses (such as arable lands, orchards and urban areas) and the Unique Agrarian Statement (DUN 2009–2013)—which provides specific information on cultivated crops and their varieties according to annual owners’ declaration. Both data were provided by the regional Department of Agriculture, Livestock, Fisheries, Food and Environment of the Generalitat de Catalunya (http://agricultura.gencat.cat). Habitat predictors were computed using ArcGIS 10.2.2 with a 1-km^2^ grid cell resolution. Human influence variables [43, 44], were also compiled considering urban areas and roads as the main ones. Terrain slope was calculated as the standard deviation of elevation in each 1-km^2^ grid cell and was derived from a digital elevation model with a resolution of 15 m.

### Spatial constraints

To account for spatially structured patterns on little bustard ranging behavior, we used spatial variables obtained through eigenvector mapping (hereafter called spatial filters; [46, 54]). Filters represent spatial aggregation at different scales in the study area [55]. Thus, their significance when they are included in distribution models indicates spatial autocorrelation in the data [46]. We computed the spatial filters in SAM 4.0 [56] by constructing a pair-wise distance matrix amongst all grid cells of the study area using their Universal Transverse Mercator coordinates (X and Y). The distance matrix was truncated at four times the maximum distance that connects all cells under minimum spanning tree criterion, and from this modified distance matrix 464 positive spatial filters were computed using principal coordinate analysis [57]. To reduce model complexity and include only relevant filters in multiple regressions assessing habitat selection patterns at the population level (see “Distribution modelling” below), we used univariable logistic models (a generalized linear model per female and year) and retained only significant filters after Bonferroni correction. This approach was used to retain potential temporal and inter-individual variation in spatial patterns in our final models. We used Moran’s I coefficients and correlograms to evaluate spatial patterns in selected filters as a measure of their spatial scale and structure [55].

### Distribution modelling

We built multiple regression models to estimate the probability of occurrence of female little bustard in relation to habitat variables and spatial filters at the population level using generalized linear mixed models (GLMMs) including data on all female and years. Female and year were included as random effects (intercepts) in those models to account for potential differences in occurrence probability. We then conducted variation partitioning analyses to separate the independent contribution of environment and spatial filters from their joint contribution (i.e., that due to spatial aggregation of occurrences related to responses to a spatially autocorrelated environment). Variation partitioning entails the calculation of incremental improvement in model fit due to the inclusion of a variable set (in our case, habitat variables and spatial filters) in models. As measure of model fit we used the deviance explained by models.

For this, we followed a hierarchical approach and ran GLMMs based on three different sets of the fixed variables, namely (1) a habitat GLMM that included only habitat variables (*HAB*); (2) a spatial GLMM that included only spatial filters (*SPAT*) and (3) a habitat and spatial GLMM that included both habitat and spatial filters (*HAB *+ *SPAT*). All continuous variables were standardized before modelling. Linear and quadratic terms of habitat variables were considered to account for non-linear relationships.

For GLMMs development, a Bernoulli error distribution for the dependent variable (presence–absence data) and a logit-link function were fitted. Absence data were generated by random selection of 50 non-used locations per female and year within the study area, which we defined as the minimum convex polygon (MCP) including the pool of presence locations of all females and years. We selected 50 locations to ensure neutral prevalence [58, 59]. To avoid model overfitting and an excess of nuisance in GLMM outputs due to the inclusion of a large number of predictors [60], we applied a backward stepwise procedure based on the Akaike Information Criterion (AIC) [61]. However full models provided highly consistent results (see “Results”). The best *HAB, SPAT* and *HAB *+ *SPAT* models were then ranked as the models receiving higher support (models with lower AIC). Dropped variables from the best *HAB* or *SPAT* models were removed and not included in more complex *HAB *+ *SPAT* models. 95% confidence intervals of fixed effect estimates were reported based on parametric bootstrap across 1000 iterations [62]. Analyses were implemented in R software (version 3.2) through ‘lme4’, ‘stats’ and ‘MuMIn’ libraries. We also evaluated the independent contribution of each variable in the *HAB, SPAT* and *HAB *+ *SPAT* models by assessing the loss in model fit (deviance explained) when dropping that predictor. Finally, to further account for potential inter-individual differences in habitat selection patterns, we re-conduct our final GLMM models by fitting an alternative random structure (random intercepts-and-slopes models) [63]. This allows estimating a random term (slope) to the coefficient of the fixed effects, so it can be different for each female. Due to model complexity, the random slope of only one fixed effect can be fitted at a time.

## Results

**Fig. 2:**
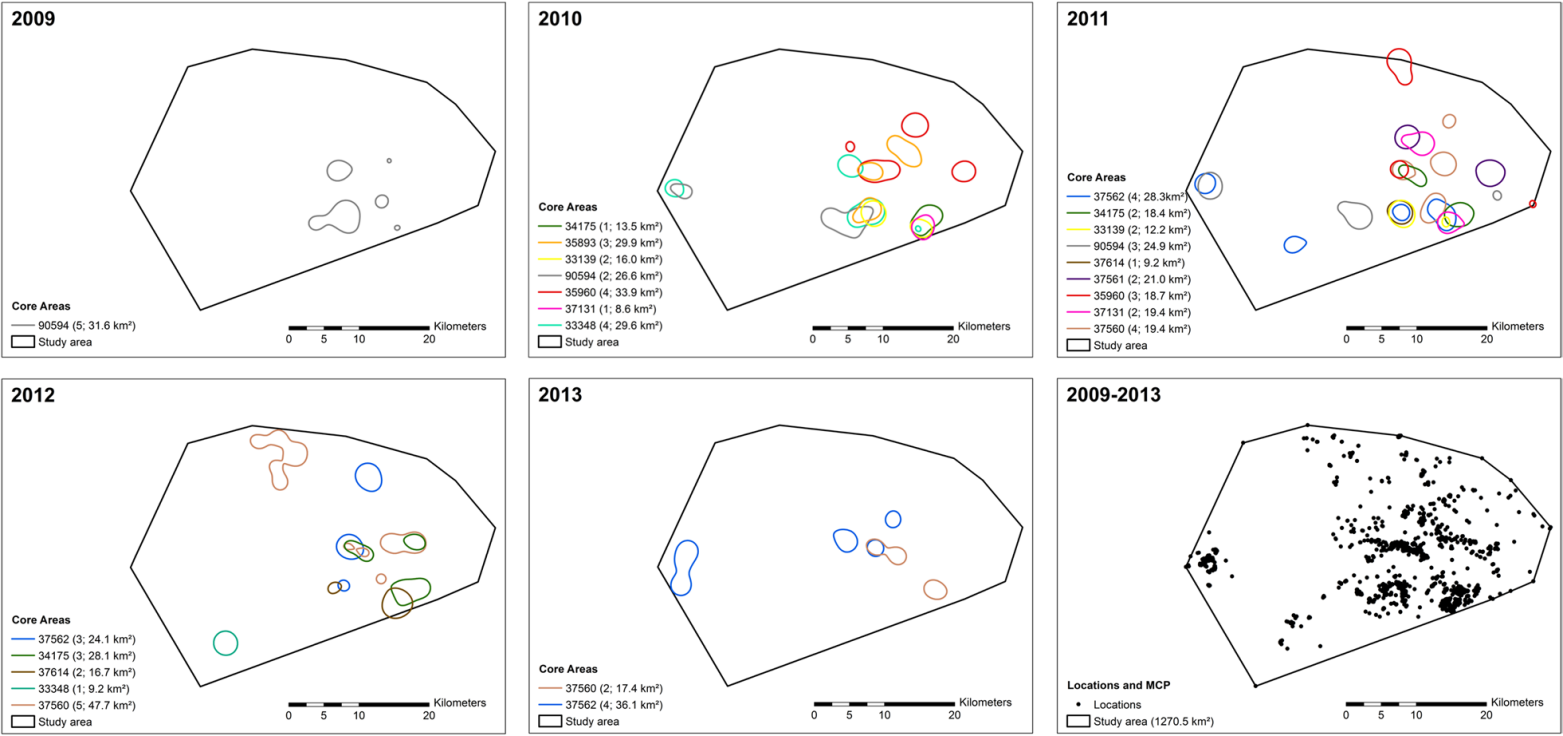
Occurrence distribution of tagged female little bustard in the study area. For graphical purposes, the core area (50% kernel) of occurrences of each female in different seasons is shown. In parenthesis, number of patches of the core area and size

In total, information on 24 non-breeding ranges was recorded from 11 females for the whole study period (3 repeated non-breeding ranges were recorded for 4 females, 2 for 5 females and one for 2 females). In different years, non-breeding ranges of 1 and 9 females were recorded. In total, female little bustard occurred in 271 grid-cells of 1 km^2^ (20%) of the study area during 2009–2013, most of them distributed in the eastern part of the study area (Fig. 2). In 86% of occupied grid cells the predominant habitat was irrigated land (> 80% of cropland), whilst in 9% it was dryland and 5% shared both habitats. The spatial overlap of presences between different females in a same year averaged 18 ± 2% (mean ± SE) and between a same female in different years averaged 25 ± 3% (mean ± SE).

### Habitat models

**Fig. 3:**
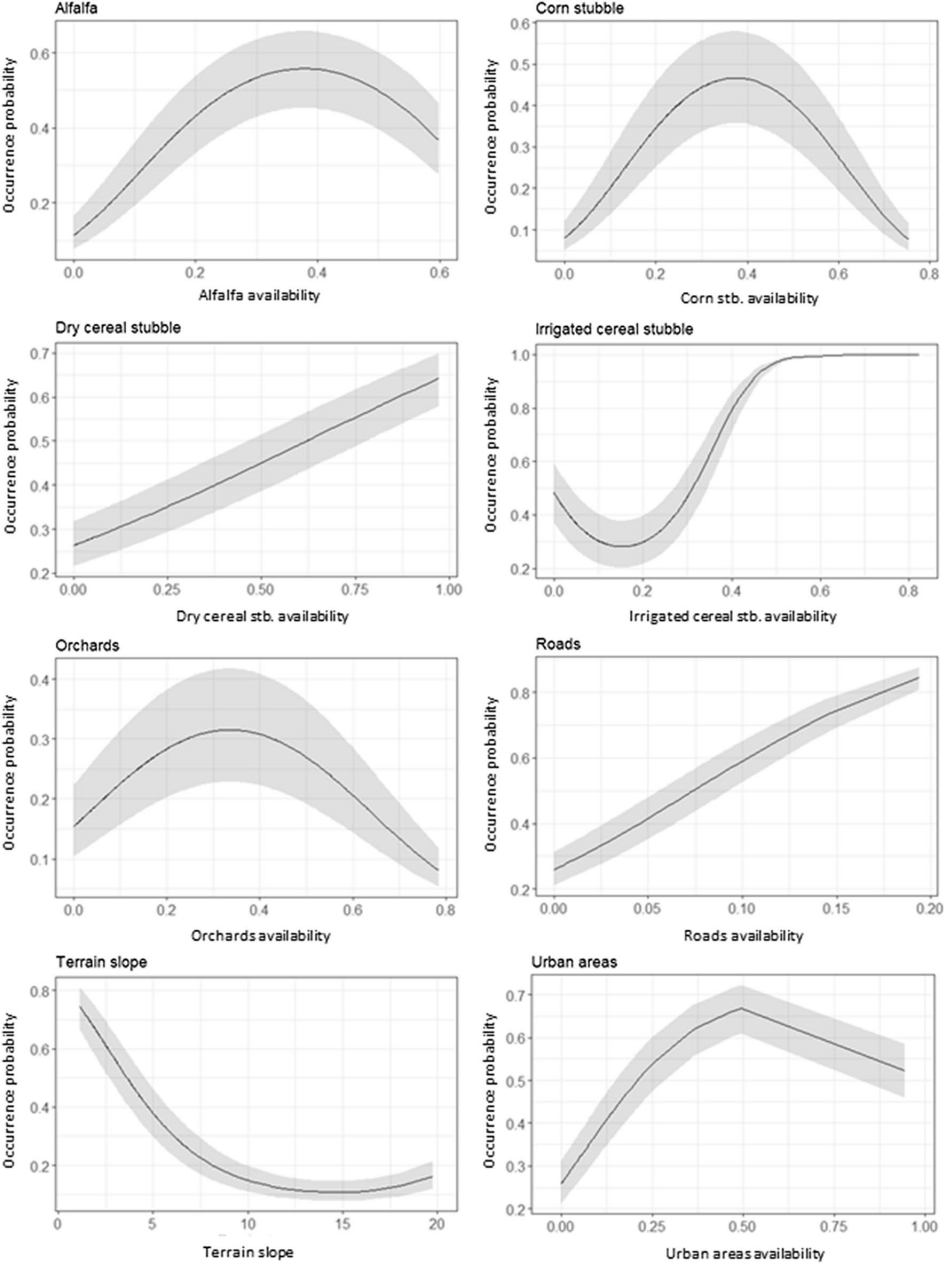
Responses curves for the best habitat model explaining the occurrence probability of female little bustard in the Plana de Lleida. Values for terrain slope response were scaled by subtracting the mean and dividing the SD

**Fig. 4:**
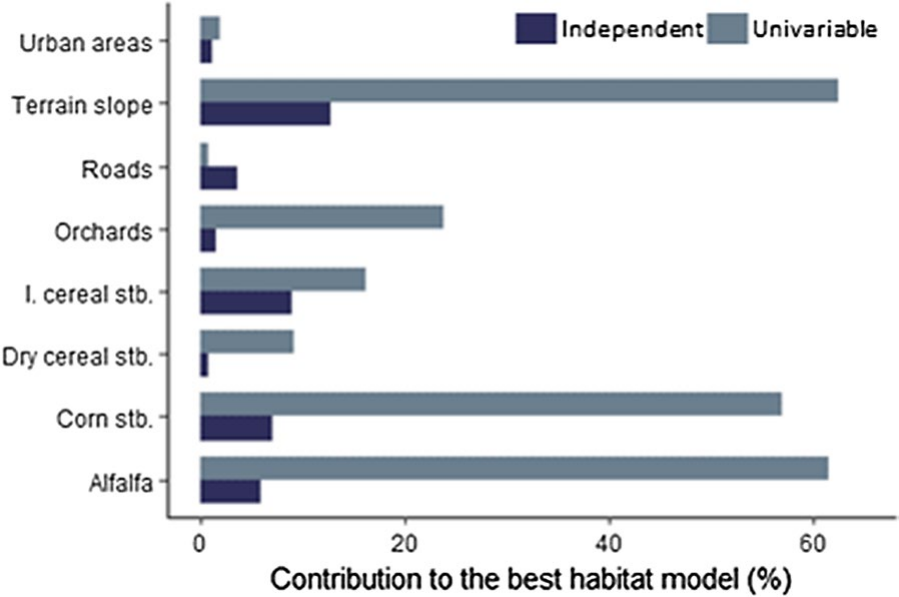
Univariable and independent contribution of habitat predictors in the best habitat model. Values are shown as percentage of the total explained variation by the best habitat model

At the population level, models revealed a non-linear negative effect of terrain slope on female occurrence probability and a positive effect of intermediate values of alfalfa and corn stubble availability (negative quadratic effect) and to small and high values of irrigated cereal stubble (positive quadratic effect) (Fig. 3, Table 2). These effects were overall consistent across different females (see Additional file 2). Female little bustard also showed a positive response to availability of roads and dry cereal stubble at the population level. However, individual response curves to such variables showed high variability among females (see Additional file 2). Attending to variable importance, slope, alfalfa, corn stubble and irrigated cereal stubble availability were the variables that most contributed to the habitat models at the population level (Fig. 4). The rest of variables showed a low independent contribution to the models (0.8–3.6% of total deviance explained by the best *HAB* model). The total percentage of deviance explained by habitat models was 35.1%.

**Table 2:** Results for the occurrence of female little bustard according to habitat models (GLMMs, logit link function)

	Best Model		Full Model	
AIC	1609.6		1613.4	
Fixed effects	β ± SE	CI	β ± SE	CI
*Intercept*	− 0.88 ± 0.13	( − 1.13, − 0.58 ) †	− 0.85 ± 0.17	( − 1.18, − 0.53 ) †
*Alfalfa*	0.98 ± 0.15	( 0.70, 1.31 ) †	1.00 ± 0.15	( 0.74, 1.38 ) †
*Alfalfa* ^*2*^	− 0.22 ± 0.06	( − 0.34, − 0.10 ) †	− 0.22 ± 0.06	( − 0.35, − 0.10 ) †
*Corn stb.*	1.10 ± 0.15	( 0.82, 1.46 ) †	1.11 ± 0.15	( 0.84, 1.46 ) †
*Corn stb.* ^*2*^	− 0.41 ± 0.07	( − 0.56, − 0.27 ) †	− 0.41 ± 0.07	( − 0.57, − 0.27 ) †
*Dry cereal stb.*	0.36 ± 0.14	( 0.09, 0.64 ) †	0.46 ± 0.30	( − 0.09, 1.09 )
*Dry cereal stb.* ^*2*^			− 0.04 ± 0.10	( − 0.24, 0.16 )
*Irrigated cereal stb.*	− 0.32 ± 0.14	( − 0.61, − 0.06 ) †	− 0.33 ± 0.14	( − 0.60, − 0.05 ) †
*Irrigated cereal stb.* ^*2*^	0.43 ± 0.06	( 0.31, 0.57 ) †	0.43 ± 0.06	( 0.32, 0.57 ) †
*Orchards*	0.34 ± 0.14	( 0.08, 0.64 ) †	0.35 ± 0.14	( 0.07, 0.66 ) †
*Orchards* ^*2*^	− 0.29 ± 0.10	( − 0.51, − 0.10 ) †	− 0.29 ± 0.10	( − 0.49, − 0.10 ) †
*Roads*	0.39 ± 0.07	( 0.26, 0.55 ) †	0.36 ± 0.16	( 0.02, 0.67 ) †
*Roads* ^*2*^			0.01 ± 0.06	( − 0.09, 0.15 )
*Terrain slope*	− 1.27 ± 0.13	( − 1.56, − 1.05 ) †	− 1.28 ± 0.13	( − 1.56, − 1.03 ) †
*Terrain slope* ^*2*^	0.33 ± 0.08	( 0.11, 0.48 ) †	0.32 ± 0.08	( 0.10, 0.47 ) †
*Urban areas*	0.68 ± 0.24	( 0.24, 1.37 ) †	0.70 ± 0.24	( 0.31, 1.37 ) †
*Urban areas* ^*2*^	− 0.07 ± 0.05	( − 0.33, − 0.00 ) †	− 0.07 ± 0.05	( − 0.35, − 0.01 ) †

The table indicates the estimates ± standard error of the variables and 95% confidence intervals generated by a bootstrap procedure (1000 iterations). Intervals not containing the zero are marked with †. Results for the model containing all the predictors and for the best model based on AIC are shown

### Spatial models

**Fig. 5:**
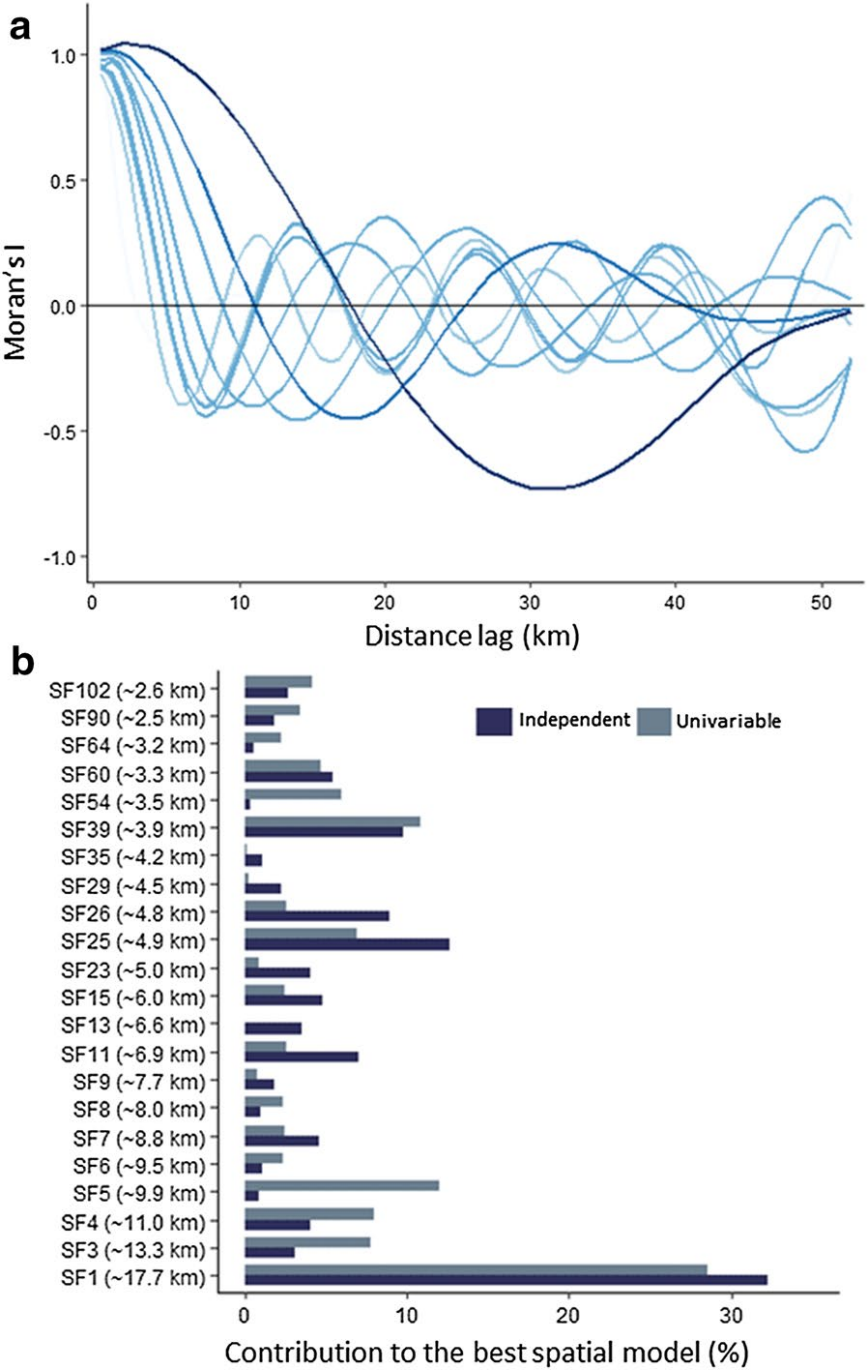
Spatial correlograms of spatial filters and their independent contribution to the best spatial model according to AIC. **a** Spatial correlograms of the 10 most important spatial filters (in order of importance: *SF1*, *SF25*, *SF39*, *SF26, SF11*, *SF60*, *SF15*, *SF7*, *SF4* and *SF23*) in spatial models defined by Moran’s I coefficients in 5 distance classes, indicating links among points of the study area successively separated by 10 km. Spatial filters are represented in a blue gradient representing filters from broad (dark) to fine scales (light). In **a** the first distance at which Moran’s I values crosses the expected value in the absence of spatial autocorrelation (0) is shown as an estimate of the scale of the spatial pattern that each filter represents. And **b**, estimate of the spatial filters in backward stepwise explaining the occurrence probability of female little bustard in the Plana de Lleida

24 spatial filters were selected to describe spatial structuration in occurrence data and be included in multiple regressions (Additional file 3). Spatial correlograms showed that selected filters reflected spatial aggregation from large (*c.* 18 km) to small (*c.* 3 km) spatial scales (Fig. 5a, see also the Additional file 4 for the map pattern of selected filters). Filters with high levels of spatial autocorrelation in the first, intermediate and last distance classes (represented by large positive and negative values of Moran’s I coefficients, Fig. 5a) tended to be portrayed by a map pattern containing few major clusters of similar values as in the first eigenvectors (e.g. *SF1*, *SF3*). As the degree of positive spatial autocorrelation decreased in filters with lower eigenvalues, the map pattern became more fragmented (e.g. *SF54*, *SF102*), representing finer-resolution spatial variation in the data (Additional file 4). Post hoc variograms representing the semi-variance in positions as a function of the time lag separating observations (Additional file 5) revealed that smaller spatial–temporal aggregation (~ 3–5 km) showed by spatial filters was consistent with spatio-temporal aggregation at low time intervals. Thus, despite birds can move as long as 23 ± 2 km per day (mean ± SE of maximum distance recorded per individual and year, n = 24), average distance traversed per day was 3.1 ± 0.2 km (mean ± SE, n = 24).

When the spatial filters were considered together in multiple regression (GLMM) representing habitat selection patterns at the population level, the spatial filter representing spatial autocorrelation at the largest scale (*SF1*) had substantially greater importance in female occurrence than other filters (Fig. 5b, see also Additional file 6 for modelling results). Other broad-scale filters (such as *SF3*, *SF4*, *SF5* and *SF6* representing spatial autocorrelation at around 10–13 km) were also important, as well as fine-scale filters such as *SF25, SF26, SF39* and *SF60* (with autocorrelation pattern at ~ 5, ~ 4 and ~ 3 km, respectively). Overall, the response of different females to the most important filters was highly consistent (Additional file 7). The total percentage of variation explained by spatial models was 81.4%.

### Habitat + spatial models

**Fig. 6:**
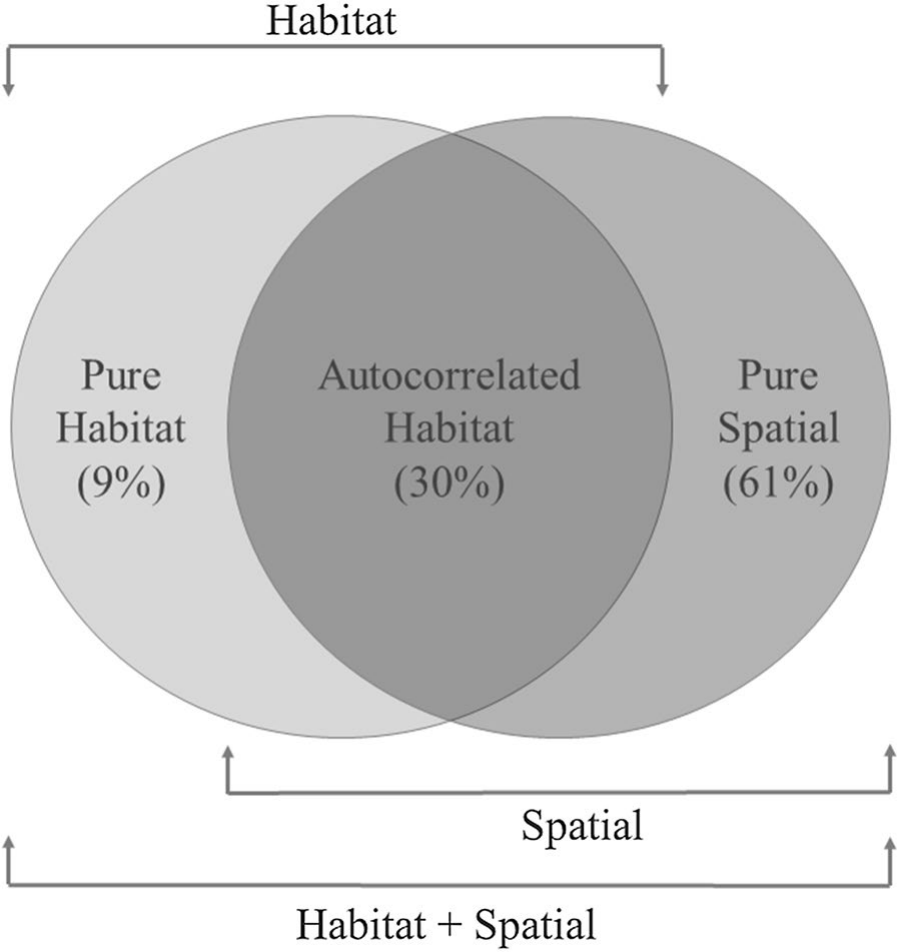
Results of the best habitat (*HAB*), spatial (*SPAT*) and more complex models combining both habitat and spatial variables (*HAB* + *SPAT*) based on stepwise AIC. Deviance partitioning analysis for the probability of occurrence of female little bustard. The figure shows a conceptual diagram of variance partitioning: the two circles represent the total variance explained by models with the two components (habitat + spatial), while the left and right circles show the variance explained by the habitat and spatial models, respectively. Percentage of total variation in occupancy rate explained by the pure and joint effects of habitat and spatial filters is shown

*HAB *+ *SPAT* models performed better than the *HAB* model. Total variation explained by the final *HAB *+ *SPAT* model was 89.2% (see Additional file 8 for the results). According to variation partitioning, pure contribution of spatial filters to variation in distribution patterns was 61%. All broad-, intermediate- and fine-scale filters contributed independently to models (Additional file 9). Independent contribution of habitat was 9% and the joint effect between habitat and spatial filters accounted for 30% (Fig. 6). Covariation between habitat and spatial variables mostly occurred at the highest scale (note the reduction in the independent effect of *SF1* in *HAB* + *SPAT *vs. *SPAT* models, Fig. 5b and Additional file 9).

## Discussion

Animals usually show non-random spatial distributions resulting from the cumulative effects of many different factors that are often difficult to separate. By using spatial eigenvector mapping in combination with habitat models, our results showed that ranging behavior of female little bustard in the non-breeding season was affected by the independent effect of habitat variables and spatial constraints, as well as by their joint effect. We found that the variance explained by the joint effect of habitat and spatial filters was high, meaning that approximately one-third of the spatial aggregation observed in female little bustard distribution was related to responses to the spatially aggregated environment. The fact that different females presented, not only spatial aggregations at the same scales, but also aggregation to the same areas of the study area, suggests that observed patterns could be related with landscape configuration heterogeneity (i.e., how different crop types are interspersed at the landscape level) in the study area. Supporting the latter, female little bustards showed a negative response to filter *SF1* (which mostly represent a decreasing western-eastern gradient, see Additional file 4). That is, female little bustards avoided the western parts of the study area, where irrigated arable land (suitable for females) appears interspersed in a more intensive farmland dominated by orchard crops (unsuitable for at least some females). Previous studies have already shown that landscape configurational heterogeneity can be an important determinant of habitat suitability perception for the species, beyond landscape composition [64].

However, a remarkable result of our research was also the high importance of pure spatial effects in the distribution models, which indicates that a good deal of the ranging behavior of female little bustards within the non-breeding season could be explained by other factors not related to habitat composition or its spatial arrangement alone. In general, the broad-scale spatial constraints representing aggregation patterns at approximately 18 km were the most important in the distribution of female little bustard. However aggregation at more local scales (~ 3–5 km) was also important. This spatial structuration may result from different, not mutually exclusive processes.

First of all, cost–benefit balances between the quality of different habitat patches and the energy required to move across them may be important. Indeed, optimal foraging theory has been widely accepted when describing foraging patterns observed in birds [65–67]. Accordingly, post hoc variograms revealed that the smaller aggregation patterns (~ 3–5 km) showed by spatial filters were consistent with spatio-temporal aggregation at low time intervals. This suggests that aggregation at local scale is mainly related with daily activity of female little bustards. Moreover, observed daily spatial structuration occurs at higher spatial scales than that reported for the breeding season [68, 69]—as it would be expected taking into account that during the non-breeding season individuals are no longer under the constraints related to reproduction and chick rearing [70].

On the second place, high concentration of little bustards in the east of the study area could be associated with the high site fidelity of the species and conspecific attraction [25, 27, 71]. Site fidelity could be important for little bustards to ensure resources (e.g. foraging places), reduce energy costs of search, and it contributes to flock aggregation. Finally, the spatial distribution of breeding and non-breeding areas within the region could partially explain the importance of spatial filters at larger scales, resulting in flocks using preferentially the areas closer to breeding sites. The distance from the occurrences’ centroids to the main breeding grounds was 14.9 ± 0.4 km (mean ± SE).

Nevertheless, even when spatial constraints largely affected female little bustard occurrences, our results also showed an independent contribution of habitat variables to observed patterns. Our explanatory models suggest that the most suitable habitat for female little bustards during the non-breeding season in the study area is flat terrains with presence of irrigated arable land. Habitat models predicted a positive effect in the occurrence probability at heterogeneous sites, with positive effects of intermediate availabilities of irrigated alfalfa and corn stubbles, as well as sites dominated by irrigated cereal stubbles. The alfalfa crops are an attractive resource for little bustards during this period, since they provide abundant food [53] and shelter [71, 72]. Additionally, recently harvested corn and cereal stubbles, holding a varied weed community, provide interesting food resources for little bustards complementary to alfalfa crops [53].

Regarding the effect of anthropogenic infrastructures, human disturbances caused by roads and urban areas have been reported as being avoided by little bustards during the breeding season [73, 74] and winter [25, 43]. Meanwhile, other studies define the species as tolerant [38, 70]. Our results suggest that the little bustard is a flexible species, with some individuals that can exhibit habituation to human disturbances in highly humanized landscapes [75]. Indeed, some females in the study area positively selected road vicinity areas. Positive effect of roads might be related to the fact that some roads are fenced and others have a high traffic intensity, which might hamper the crossing of predators or people, which otherwise may kill or simply flush the birds [76, 77]. Additionally, in the study area roads are provided with a hunting security band where shooting is totally banned, which may act as a refuge for the species, subject to some degree of illegal killing pressure [34, 78]. In that way, areas in the vicinity of certain roads may provide relatively quiet places, which could be selected by some little bustards, while other individuals might be more sensitive to stress levels produced by anthropogenic disturbances [79, 80].

## Conclusions

Altogether, the present study increases our understanding of habitat selection patterns of a threatened steppe bird species and their associated spatial patterns at a regional scale during the non-breeding season. Individual responses to habitat and spatial variables provide evidence of a high inter-individual consistency in overall habitat selection patterns. According to such results, measures to promote flat open heterogeneous landscapes, with alfalfa and stubble availability, in safe places as close as possible to breeding sites, could contribute to benefit resident or scarcely migratory little bustard populations in non-breeding grounds. Our results also highlight the need to investigate further about the role of roads on the ranging behavior of this and other species occupying highly humanized landscapes. Although the specific process underlying the observed selection of roads is not yet well understood, promoting areas with restricted game shooting and limiting human access may help to improve the quality of the non-breeding grounds and, consequently, the condition of the birds. Finally, our results show the existence of spatial constraints in occurrence patterns beyond landscape composition. Whether site fidelity and conspecific attraction and/or configurational heterogeneity are involved in observed patterns must be addressed to anticipate potential effects of new land-use changes and provide timely advice toward long-term management planning.

## Additional files

**Additional file 1.** Flock association between female little bustards in a same year. Pairwise estimates based on HWI index.

**Additional file 2.** Random intercepts-and-slopes habitat models. Variation in habitat predictors of the best habitat model within the female factor.

**Additional file 3.** Retained spatial filters. Number of times that spatial filters were retained in univariable spatial models for each female and year.

**Additional file 4.** Map patterns of spatial filters. Geographic patterns of the spatial filters included in univariable models testing occurrence probability of female little bustards in the non-breeding season.

**Additional file 5.** Variograms of distance between positions. Variograms representing the average square distance between positions (semi-variance) as a function of the time lag separating observations.

**Additional file 6.** Results of spatial models (*SPAT*). Estimates of fixed effects predicting the occurrence probability of female little bustard according to spatial models.

**Additional file 7.** Random intercepts-and-slopes spatial models. Variation in spatial predictors of the best spatial model within the female factor.

**Additional file 8.** Results of *HAB *+ *SPAT* models. Estimates of fixed effects predicting the occurrence probability of female little bustard according to *SPAT *+ *HAB* models.

**Additional file 9.** Contribution of predictors to *HAB *+ *SPAT* models. Independent contribution of different predictors to the best *HAB *+ *SPAT* model.

## Abbreviations

AIC: Akaike Information Criterion
DUN: unique agrarian statement
GIS: geographic information system
GLMM: generalized linear mixed models
GMT: Greenwich Mean Time
HAB: habitat models
HAB + SPAT: habitat and spatial models
SF: spatial filter
SPAT: spatial models
UTM: Universal Transverse Mercator
