# Supplementary material for: Inter-individual consistency in habitat selection patterns and spatial range constraints of female little bustards during the non-breeding season
Source: BMC Ecol. 2018 Dec 5;18:56. doi: 10.1186/s12898-018-0205-9 (PMC6280389; doi:10.1186/s12898-018-0205-9)
Supplement: Supplementary file 1 — Additional file 1. Flock association between female little bustards in a same year. Pairwise estimates based on HWI index. [file 12898_2018_205_MOESM1_ESM.docx]

**Additional file 1**

Flocking

As the little bustard is a gregarious species during non-breeding season (Cramp and Simmons 1980), data can be highly aggregated among individuals. To account for possible non-independence of the data, we calculated pairwise association of females in a same flock in different years. To obtain female association we used the Half-Weight Index (HWI) (Cairns and Schwager 1987). This index measure association degree between two individuals and it can be expressed as: HWI_ab_ = *x* / ½ (*y*_a_ + *y*_b_), where *x* is the number of times in which two females (*a* and *b*) were associated in a same flock, and *y*_a_ and *y*_b_ correspond to the total of locations for the female *a* and for the female *b*. This index ranges from 0 (two females never shared a flock) and 1 (two females always detected together). Two females were considered to be in a same flock whether at time *t* they were located at < 500 m distance. The flock association (HWI) between females was relatively low (0.04 ± 0.06; mean ± SD), where in the 50% of pairwise comparisons HWI showed a value equals to 0 and in the 38% was below 0.1 (see the upper hemi-matrices in Table S1).

Cairns, S. J., and S. J. Schwager (1987). A comparison of association indices. Animal Behaviour 35:1454–1469. doi: 10.1016/S0003-3472(87)80018-0

Cramp, S., and K. E. L. Simmons (1980). The Birds of the Western Palearctic. In. Oxford.

**Table S1.** Results for the flock association between female little bustards (HWI: Half-Weight Index) in a same year.

| ***Year*** | ***Female*** | **90594** |  |  |  |  |  |  |  |  |  |
| --- | --- | --- | --- | --- | --- | --- | --- | --- | --- | --- | --- |
| **2009** | **90594** | - |  |  |  |  |  |  |  |  |  |
|  |  |  |  |  |  |  |  |  |  |  |  |
| ***Year*** | ***Female*** | **34175** | **35893** | **33139** | **90594** | **35960** |  | **37131** | **33348** |  |  |
| **2010** | **34175** | - | 0.08 | 0.15 | 0.00 | 0.00 |  | 0.18 | 0.02 |  |  |
|  | **35893** |  | - | 0.02 | 0.06 | 0.03 |  | 0.00 | 0.15 |  |  |
|  | **33139** |  |  | - | 0.01 | 0.00 |  | 0.23 | 0.03 |  |  |
|  | **90594** |  |  |  | - | 0.00 |  | 0.00 | 0.15 |  |  |
|  | **35960** |  |  |  |  | - |  | 0.00 | 0.00 |  |  |
|  | **37131** |  |  |  |  |  |  | - | 0.01 |  |  |
|  | **33348** |  |  |  |  |  |  |  | - |  |  |
|  |  |  |  |  |  |  |  |  |  |  |  |
| ***Year*** | ***Female*** | **37562** | **34175** | **33139** | **90594** | **37614** |  | **37561** | **35960** | **37131** | **37560** |
| **2011** | **37562** | - | 0.04 | 0.15 | 0.00 | 0.09 |  | 0.00 | 0.00 | 0.00 | 0.09 |
|  | **34175** |  | - | 0.04 | 0.00 | 0.03 |  | 0.02 | 0.00 | 0.01 | 0.04 |
|  | **33139** |  |  | - | 0.00 | 0.33 |  | 0.05 | 0.00 | 0.07 | 0.00 |
|  | **90594** |  |  |  | - | 0.00 |  | 0.00 | 0.00 | 0.00 | 0.00 |
|  | **37614** |  |  |  |  | - |  | 0.00 | 0.00 | 0.03 | 0.00 |
|  | **37561** |  |  |  |  |  |  | - | 0.06 | 0.00 | 0.05 |
|  | **35960** |  |  |  |  |  |  |  | - | 0.00 | 0.03 |
|  | **37131** |  |  |  |  |  |  |  |  | - | 0.00 |
|  | **37560** |  |  |  |  |  |  |  |  |  | - |
|  |  |  |  |  |  |  |  |  |  |  |  |
| ***Year*** | ***Female*** | **37562** | **34175** | **37614** | **33348** | **37560** |  |  |  |  |  |
| **2012** | **37562** | - | 0.01 | 0.00 | 0.02 | 0.00 |  |  |  |  |  |
|  | **34175** |  | - | 0.01 | 0.00 | 0.14 |  |  |  |  |  |
|  | **37614** |  |  | - | 0.00 | 0.01 |  |  |  |  |  |
|  | **33348** |  |  |  | - | 0.00 |  |  |  |  |  |
|  | **37560** |  |  |  |  | - |  |  |  |  |  |
|  |  |  |  |  |  |  |  |  |  |  |  |
| ***Year*** | ***Female*** | **37562** | **37560** |  |  |  |  |  |  |  |  |
| **2013** | **37562** | - | 0.00 |  |  |  |  |  |  |  |  |
|  | **37560** |  | - |  |  |  |  |  |  |  |  |
