# Supplementary material for: Inter-individual consistency in habitat selection patterns and spatial range constraints of female little bustards during the non-breeding season
Source: BMC Ecol. 2018 Dec 5;18:56. doi: 10.1186/s12898-018-0205-9 (PMC6280389; doi:10.1186/s12898-018-0205-9)
Supplement: Supplementary file 3 — Additional file 3. Retained spatial filters. Number of times that spatial filters were retained in univariable spatial models for each female and year. [file 12898_2018_205_MOESM3_ESM.docx]

**Additional file 3**

**Fig S3** Spatial filters retained to be included in multivariate regression (N=24). Number of times that each spatial filter was retained in univariable spatial models conducted for each female and year.

**
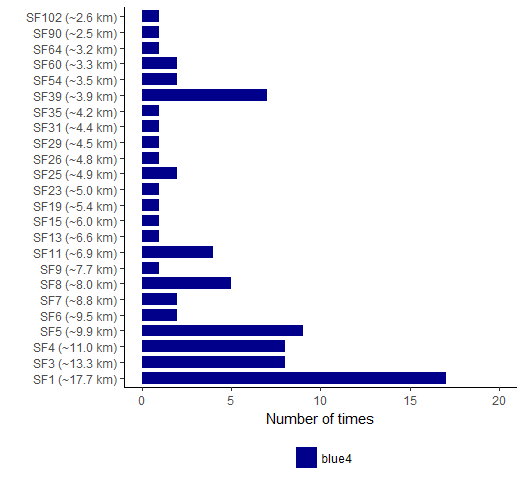
**
