# Supplementary material for: Inter-individual consistency in habitat selection patterns and spatial range constraints of female little bustards during the non-breeding season
Source: BMC Ecol. 2018 Dec 5;18:56. doi: 10.1186/s12898-018-0205-9 (PMC6280389; doi:10.1186/s12898-018-0205-9)

Additional file 4

Figure S4 Geographic patterns of 24 spatial filters resulting significant in univariate test for occurrence distribution of at least one female and non-breeding season. Positive values are represented in a dark gradient and negative values in clear.

SF1

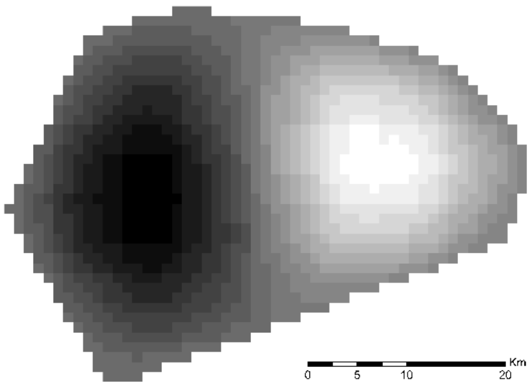

SF3

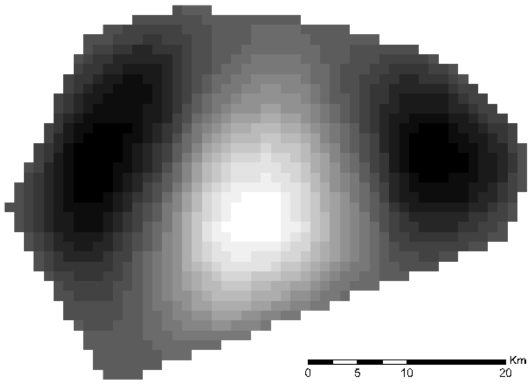

SF4

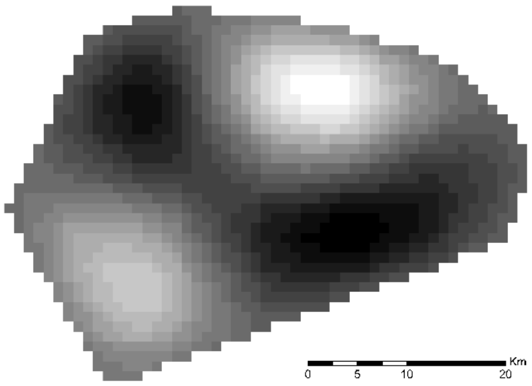

SF5

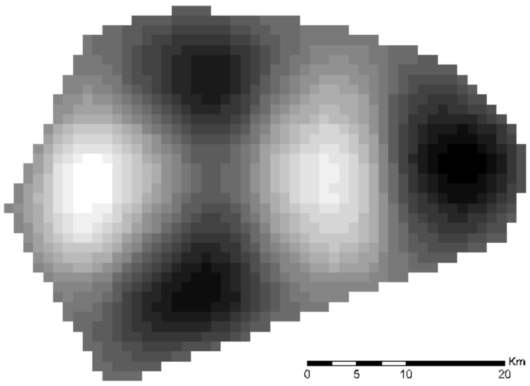

SF6

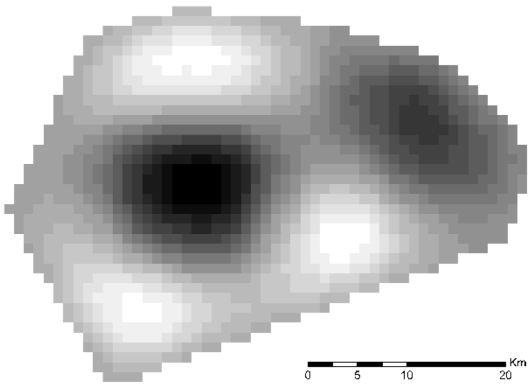

SF7

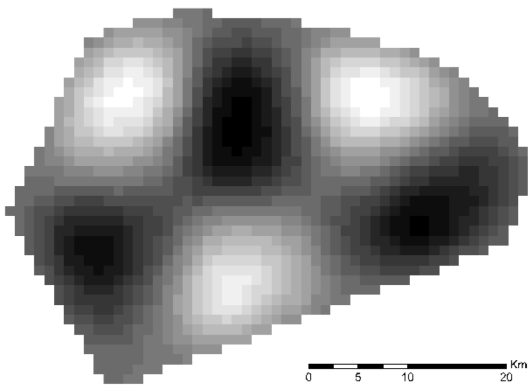

SF8

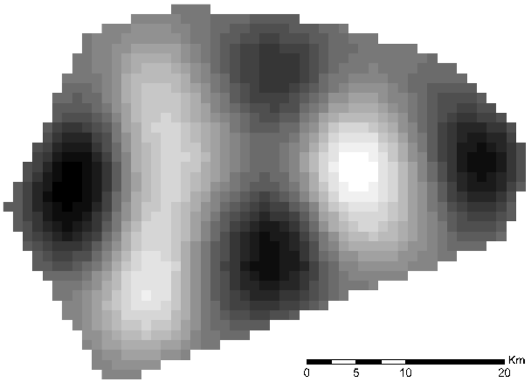

SF9

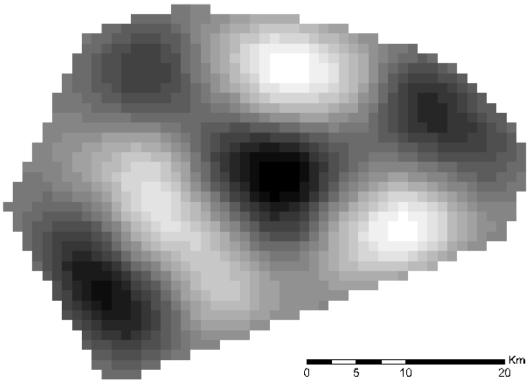

SF11

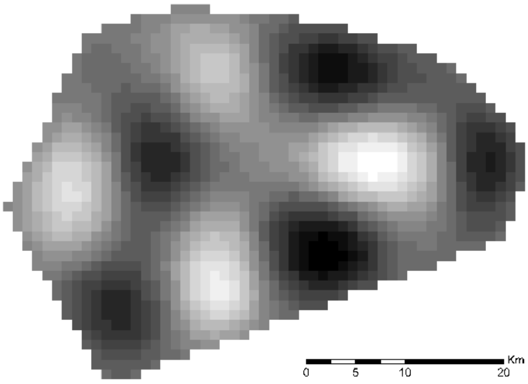

SF13

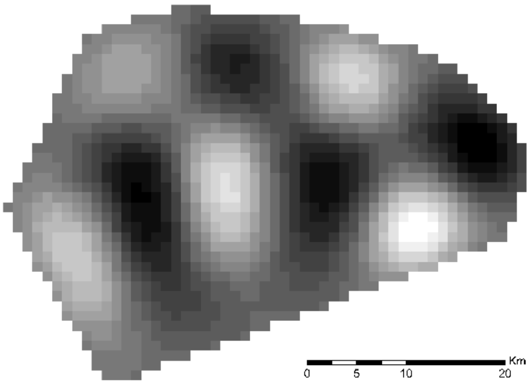

SF15

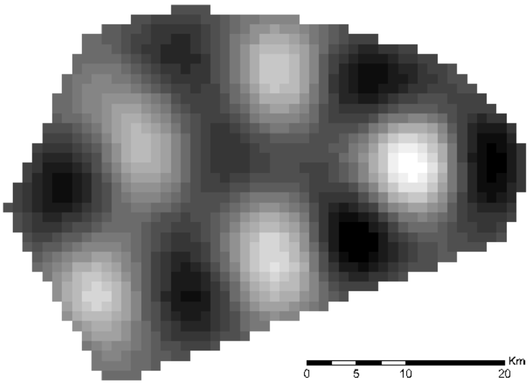

SF19

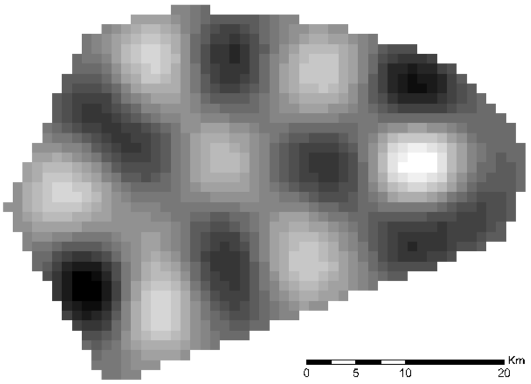

SF23

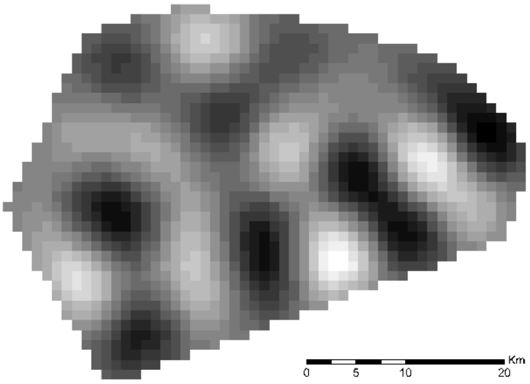

SF25

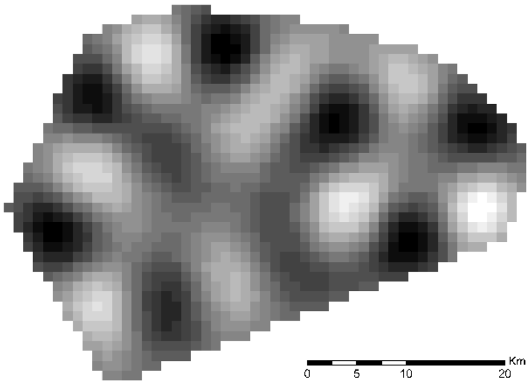

SF26

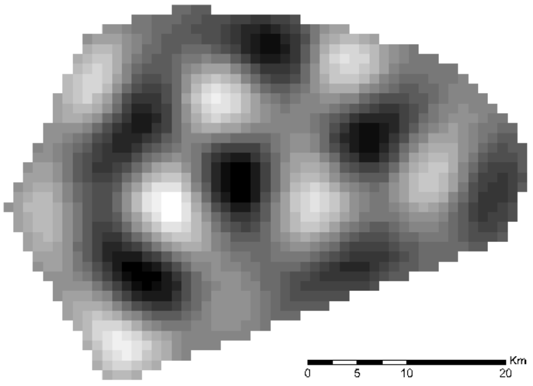

SF29

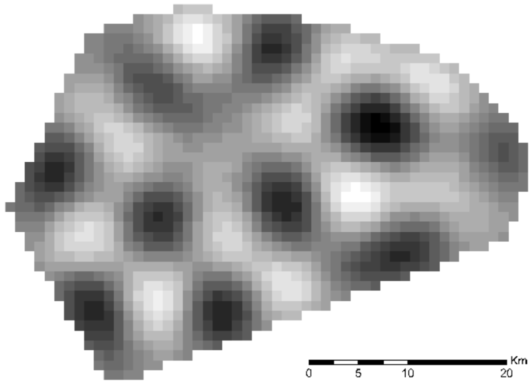

SF31

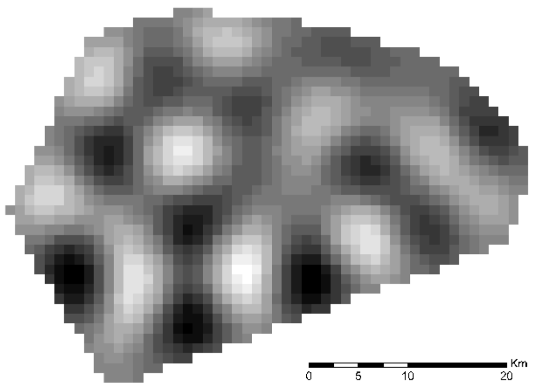

SF35

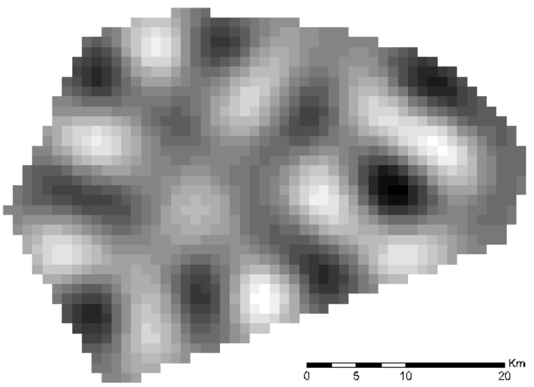

SF39

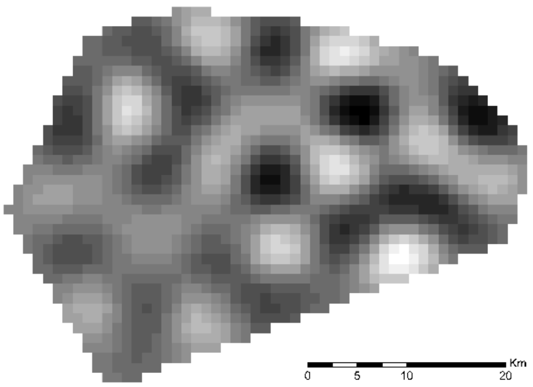

SF54

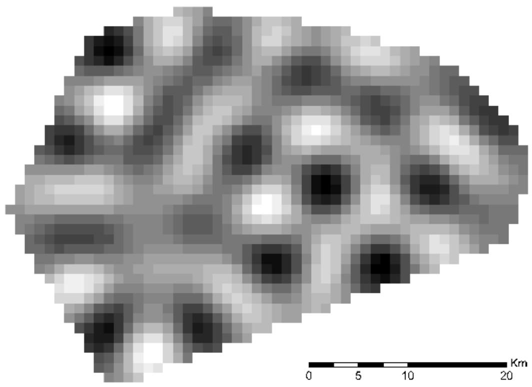

SF60

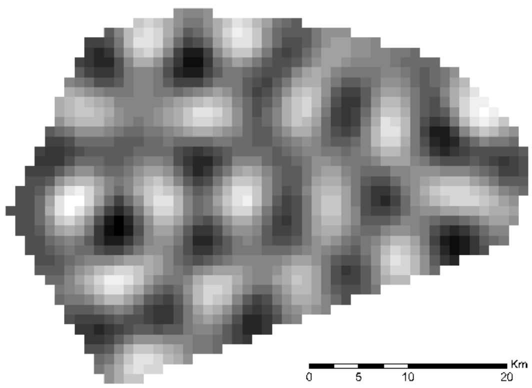

SF64

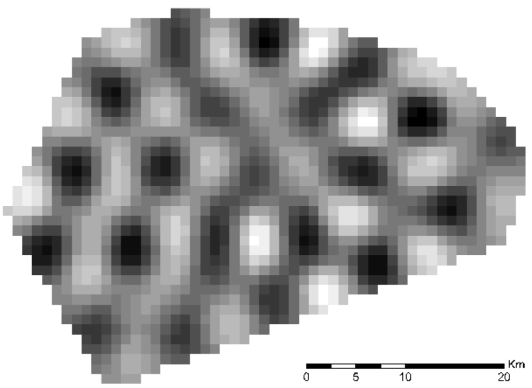

SF90

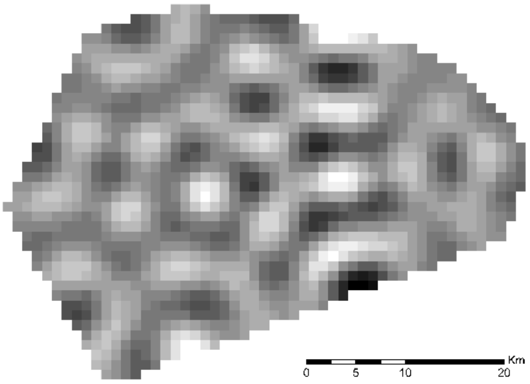

SF102

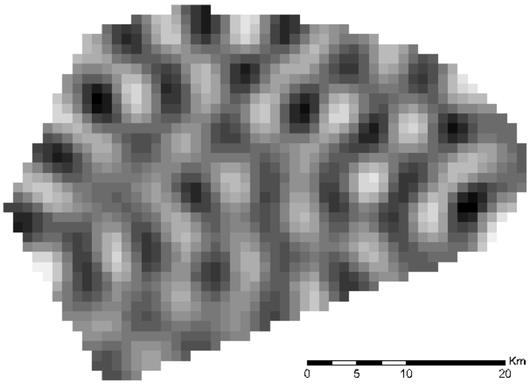

Supplement: Supplementary file 4 — Additional file 4. Map patterns of spatial filters. Geographic patterns of the spatial filters included in univariable models testing occurrence probability of female little bustards in the non-breeding season. [file 12898_2018_205_MOESM4_ESM.pdf]
