# Supplementary material for: Inter-individual consistency in habitat selection patterns and spatial range constraints of female little bustards during the non-breeding season
Source: BMC Ecol. 2018 Dec 5;18:56. doi: 10.1186/s12898-018-0205-9 (PMC6280389; doi:10.1186/s12898-018-0205-9)
Supplement: Supplementary file 5 — Additional file 5. Variograms of distance between positions. Variograms representing the average square distance between positions (semi-variance) as a function of the time lag separating observations. [file 12898_2018_205_MOESM5_ESM.docx]

**Additional file 5**

**
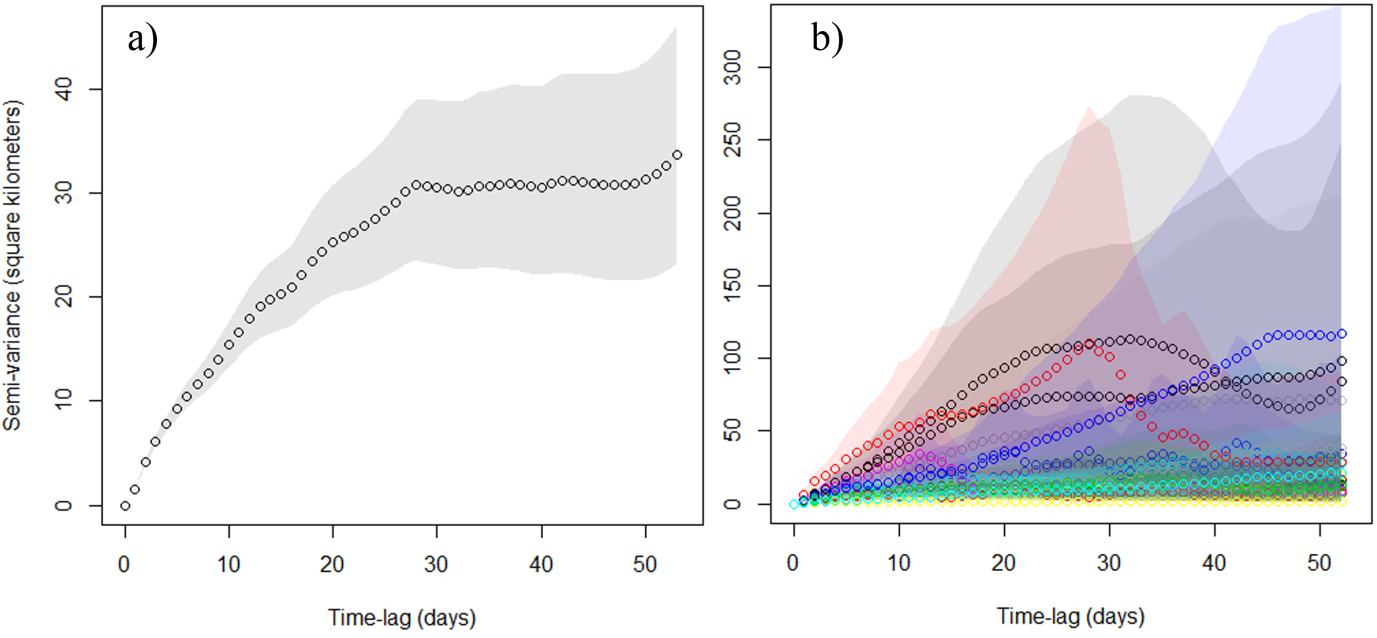
Fig S5** Variograms representing the average square distance between positions (semi-variance) as a function of the time lag separating observations. Mean semi-variance values across all individuals and years (a) and individual values (b) are shown. Shadow areas represent 95% confidence intervals around the semi-variance estimates.
