# Supplementary material for: Inter-individual consistency in habitat selection patterns and spatial range constraints of female little bustards during the non-breeding season
Source: BMC Ecol. 2018 Dec 5;18:56. doi: 10.1186/s12898-018-0205-9 (PMC6280389; doi:10.1186/s12898-018-0205-9)
Supplement: Supplementary file 6 — Additional file 6. Results of spatial models (SPAT). Estimates of fixed effects predicting the occurrence probability of female little bustard according to spatial models. [file 12898_2018_205_MOESM6_ESM.docx]

| **Addtional file 6**  **Table S6.** Results for the occurrence of female little bustard according to spatial models (GLMMs, logit link function). The table indicates the estimates ± standard error and 95% confidence intervals generated by bootstrap procedure (1000 iterations). Intervals not containing the zero are marked with †. Results for the model containing all the predictors and for the best model based on AIC are shown. | | | | | |
| --- | --- | --- | --- | --- | --- |
|  |  |  |  |  |  |
|  |  |  |  |  |  |
|  |  |  |  |  |  |
|  | **Best Model** | |  | **Full Model** | |
| **AIC** | **502.2** | |  | **504.7** | |
| **Fixed effects** | ** β ± SE** | **CI** |  | ** β ± SE** | **CI** |
| *Intercept* | 0.78 ± 0.50 | ( −0.16, 2.02 ) |  | 0.70 ± 0.51 | ( −0.33, 2.02 ) |
| *SF1* | −8.45 ± 0.92 | ( −11.69, −7.23 ) † |  | −8.98 ± 1.08 | ( −13.06, −7.80 ) † |
| *SF3* | −2.11 ± 0.30 | ( −3.01, −1.60 ) † |  | −2.39 ± 0.39 | ( −3.70, −1.83 ) † |
| *SF4* | 2.26 ± 0.32 | ( 1.79, 3.29 ) † |  | 2.23 ± 0.33 | ( 1.73, 3.22 ) † |
| *SF5* | −1.75 ± 0.47 | ( −2.97, −0.95 ) † |  | −1.45 ± 0.53 | ( −2.80, −0.55 ) † |
| *SF6* | −1.63 ± 0.38 | ( −2.93, −1.02 ) † |  | −1.99 ± 0.50 | ( −3.80, −1.19 ) † |
| *SF7* | 1.98 ± 0.25 | ( 1.61, 2.84 ) † |  | 2.05 ± 0.27 | ( 1.72, 3.07 ) † |
| *SF8* | −1.61 ± 0.37 | ( −2.58, −0.93 ) † |  | −1.69 ± 0.41 | ( −2.82, −1.04 ) † |
| *SF9* | −2.06 ± 0.39 | ( −3.11, −1.42 ) † |  | −2.07 ± 0.41 | ( −3.26, −1.45 ) † |
| *SF11* | −4.34 ± 0.60 | ( −6.25, −3.52 ) † |  | −4.37 ± 0.66 | ( −6.53, −3.65 ) † |
| *SF13* | −2.21 ± 0.32 | ( −3.21, −1.72 ) † |  | −2.33 ± 0.35 | ( −3.50, −1.86 ) † |
| *SF15* | 2.34 ± 0.33 | ( 1.92, 3.37 ) † |  | 2.40 ± 0.36 | ( 1.96, 3.58 ) † |
| *SF19* |  |  |  | 0.29 ± 0.33 | ( −0.33, 1.07 ) |
| *SF23* | 2.69 ± 0.40 | ( 2.14, 3.93 ) † |  | 2.67 ± 0.46 | ( 2.05, 4.19 ) † |
| *SF25* | 4.78 ± 0.51 | ( 4.11, 6.44 ) † |  | 5.11 ± 0.61 | ( 4.47, 7.40 ) † |
| *SF26* | 3.44 ± 0.40 | ( 2.95, 4.73 ) † |  | 3.54 ± 0.42 | ( 3.07, 5.04 ) † |
| *SF29* | −1.65 ± 0.29 | ( −2.52, −1.21 ) † |  | −1.75 ± 0.31 | ( −2.71, −1.30 ) † |
| *SF31* |  |  |  | −0.26 ± 0.32 | ( −1.08, 0.42 ) |
| *SF35* | 1.30 ± 0.31 | ( 0.76, 2.13 ) † |  | 1.33 ± 0.35 | ( 0.73, 2.36 ) † |
| *SF39* | −3.35 ± 0.38 | ( −4.63, −2.87 ) † |  | −3.49 ± 0.43 | ( −5.08, −3.01 ) † |
| *SF54* | −0.89 ± 0.38 | ( −1.80, −0.21 ) † |  | −0.95 ± 0.39 | ( −1.88, −0.21 ) † |
| *SF60* | −2.29 ± 0.28 | ( −3.11, −1.90 ) † |  | −2.31 ± 0.28 | ( −3.26, −1.92 ) † |
| *SF64* | 0.60 ± 0.20 | ( 0.23, 1.10 ) † |  | 0.57 ± 0.20 | ( 0.19, 1.12 ) † |
| *SF90* | −1.23 ± 0.23 | ( −1.82, −0.87 ) † |  | −1.20 ± 0.23 | ( −1.82, −0.83 ) † |
| *SF102* | −1.51 ± 0.24 | ( −2.17, −1.10 ) † |  | −1.60 ± 0.27 | ( −2.47, −1.24 ) † |
