# Supplementary material for: Inter-individual consistency in habitat selection patterns and spatial range constraints of female little bustards during the non-breeding season
Source: BMC Ecol. 2018 Dec 5;18:56. doi: 10.1186/s12898-018-0205-9 (PMC6280389; doi:10.1186/s12898-018-0205-9)

Additional file 7

<u>Inter-individual variation</u>

Fig S7 Partial response curves showing individual responses of female little bustard to spatial predictors based on random intercepts-and-slopes models.

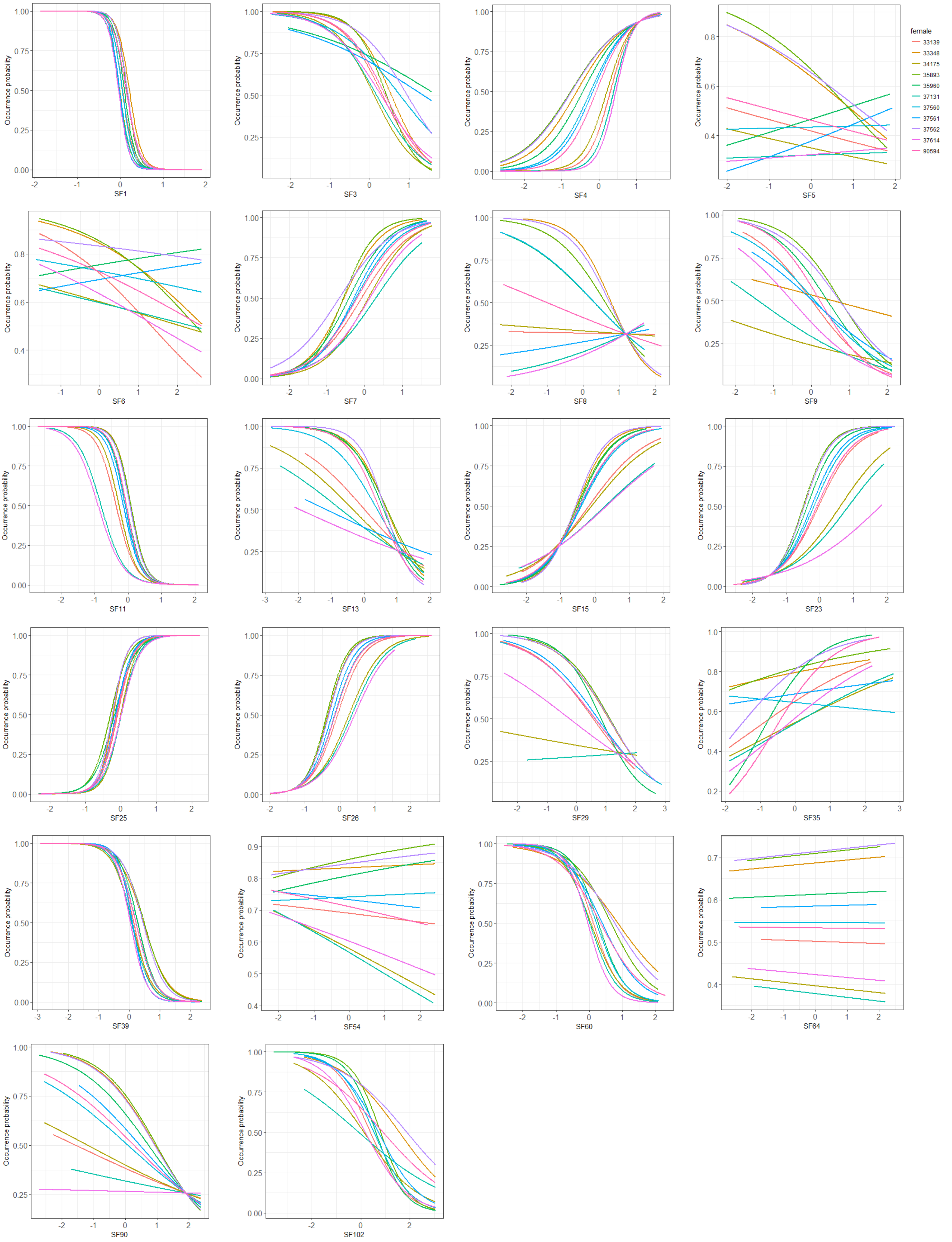

Supplement: Supplementary file 7 — Additional file 7. Random intercepts-and-slopes spatial models. Variation in spatial predictors of the best spatial model within the female factor. [file 12898_2018_205_MOESM7_ESM.pdf]
