# Supplementary material for: Inter-individual consistency in habitat selection patterns and spatial range constraints of female little bustards during the non-breeding season
Source: BMC Ecol. 2018 Dec 5;18:56. doi: 10.1186/s12898-018-0205-9 (PMC6280389; doi:10.1186/s12898-018-0205-9)
Supplement: Supplementary file 8 — Additional file 8. Results of HAB + SPAT models. Estimates of fixed effects predicting the occurrence probability of female little bustard according to SPAT + HAB models. [file 12898_2018_205_MOESM8_ESM.docx]

**Additional file 8**

| **Table S8.** Results for the occurrence of female little bustard according to habitat+spatial models (GLMMs, logit link function). The table indicates the estimates ± standard error and 95% confidence intervals generated by bootstrap procedure (1000 iterations). Intervals not containing the zero are marked with †. Results for the model containing all the predictors and for the best model based on AIC are shown. | | | | | |
| --- | --- | --- | --- | --- | --- |
|  | **Best Model** | |  | **Full Model** | |
| **AIC** | **331.1** | |  | **338.4** | |
| **Fixed effects** | ** β ± SE** | **CI** |  | ** β ± SE** | **CI** |
| *Intercept* | −2.32 ± 0.84 | ( −5.52, 0.10 ) |  | −2.68 ± 0.97 | ( −6.96, 1.15 ) |
| *Alfalfa* | 0.87 ± 0.51 | ( −0.31, 2.63 ) |  | 0.92 ± 0.53 | ( −0.36, 2.93 ) |
| *Alfalfa^2^* | −0.44 ± 0.20 | ( −1.09, −0.01 ) † |  | −0.44 ± 0.20 | ( −1.21, 0.02 ) |
| *Corn stb.* | −2.15 ± 0.77 | ( −4.99, −0.46 ) † |  | −2.04 ± 0.80 | ( −5.16, −0.23 ) † |
| *Corn stb.^2^* | −0.57 ± 0.28 | ( −1.54, −0.04 ) † |  | −0.60 ± 0.29 | ( −1.74, −0.03 ) † |
| *Dry cereal stb.* | −4.17 ± 0.72 | ( −7.74, −2.94 ) † |  | −4.33 ± 0.77 | ( −8.36, −3.27 ) † |
| *Irrigated cereal stb.* | −3.71 ± 0.54 | ( −6.41, −3.05 ) † |  | −3.68 ± 0.58 | ( −6.93, −2.92 ) † |
| *Irrigated cereal stb.^2^* | 1.78 ± 0.31 | ( 1.38, 3.30 ) † |  | 1.76 ± 0.31 | ( 1.36, 3.47 ) † |
| *Orchards* | −1.63 ± 0.58 | ( −3.83, −0.14 ) † |  | −1.73 ± 0.65 | ( −4.27, 0.11 ) |
| *Orchards^2^* | 0.60 ± 0.42 | ( −0.43, 2.16 ) |  | 0.64 ± 0.43 | ( −0.58, 2.38 ) |
| *Roads* |  |  |  | 0.13 ± 0.36 | ( −0.84, 1.41 ) |
| *Terrain slope* | −6.44 ± 0.89 | ( −12.17, −5.68 ) † |  | −6.69 ± 1.00 | ( −13.82, −5.95 ) † |
| *Terrain slope^2^* | 2.94 ± 0.46 | ( 2.36, 5.61 ) † |  | 3.08 ± 0.52 | ( 2.41, 6.32 ) † |
| *Urban areas* | −8.91 ± 1.70 | ( −16.03, −5.27 ) † |  | −9.42 ± 1.79 | ( −18.09, −5.50 ) † |
| *Urban areas^2^* | 1.55 ± 0.45 | ( 0.14, 2.82 ) † |  | 1.66 ± 0.41 | ( 0.15, 3.33 ) † |
| *SF1* | −11.54 ± 1.45 | ( −22.78, −10.83 ) † |  | −11.59 ± 1.51 | ( −26.90, −11.13 †) |
| *SF3* | −3.68 ± 0.59 | ( −7.27, −3.10 ) † |  | −3.66 ± 0.62 | ( −8.35, −2.96 ) † |
| *SF4* | 5.10 ± 0.63 | ( 4.57, 9.33 ) † |  | 5.16 ± 0.66 | ( 4.78, 10.33 ) † |
| *SF5* |  |  |  | 0.37 ± 0.65 | ( −2.88, 2.58 ) |
| *SF6* | −5.49 ± 0.74 | ( −11.32, −4.89 ) † |  | −5.90 ± 0.97 | ( −13.55, −4.94 ) † |
| *SF7* | 2.64 ± 0.37 | ( 2.29, 5.35 ) † |  | 2.62 ± 0.37 | ( 2.27, 6.26 ) † |
| *SF8* | −3.62 ± 0.71 | ( −7.09, −1.98 ) † |  | −3.59 ± 0.74 | ( −8.04, −2.06 ) † |
| *SF9* | −3.43 ± 0.51 | ( −6.90, −2.55 ) † |  | −3.41 ± 0.56 | ( −7.94, −2.63 ) † |
| *SF11* | −5.85 ± 0.80 | ( −11.83, −5.29 ) † |  | −5.82 ± 0.86 | ( −13.80, −5.55 ) † |
| *SF13* | −4.27 ± 0.52 | ( −8.30, −4.00 ) † |  | −4.47 ± 0.61 | ( −9.74, −4.15 ) † |
| *SF15* | 4.71 ± 0.62 | ( 4.33, 9.06 ) † |  | 4.68 ± 0.65 | ( 4.47, 10.34 ) † |
| *SF23* | 3.41 ± 0.58 | ( 2.80, 7.13 ) † |  | 3.35 ± 0.60 | ( 2.91, 8.26 ) † |
| *SF25* | 8.75 ± 1.09 | ( 8.06, 16.02 ) † |  | 8.90 ± 1.11 | ( 8.44, 18.96 ) † |
| *SF26* | 5.77 ± 0.75 | ( 5.23, 10.55 ) † |  | 5.89 ± 0.80 | ( 5.46, 12.85 ) † |
| *SF29* | −2.26 ± 0.49 | ( −4.60, −1.58 ) † |  | −2.24 ± 0.54 | ( −5.44, −1.57 ) † |
| *SF35* | 1.84 ± 0.53 | ( 0.79, 4.10 ) † |  | 2.01 ± 0.59 | ( 0.83, 4.89 ) † |
| *SF39* | −6.24 ± 0.83 | ( −11.68, −5.73 ) † |  | −6.21 ± 0.84 | ( −13.61, −5.77 ) † |
| *SF54* |  |  |  | −0.16 ± 0.52 | ( −2.37, 1.37 ) |
| *SF60* | −2.57 ± 0.38 | ( −4.93, −2.20 ) † |  | −2.53 ± 0.40 | ( −5.92, −2.16 ) † |
| *SF64* |  |  |  | −0.12 ± 0.36 | ( −1.39, 1.12 ) |
| *SF90* | −0.76 ± 0.38 | ( −2.28, −0.03 ) † |  | −0.65 ± 0.41 | ( −2.76, 0.25 ) |
| *SF102* | −3.18 ± 0.44 | ( −5.96 ± −2.81 ) † |  | −3.34 ± 0.50 | ( −6.90 ± −3.01 ) † |
