# Supplementary material for: Inter-individual consistency in habitat selection patterns and spatial range constraints of female little bustards during the non-breeding season
Source: BMC Ecol. 2018 Dec 5;18:56. doi: 10.1186/s12898-018-0205-9 (PMC6280389; doi:10.1186/s12898-018-0205-9)
Supplement: Supplementary file 9 — Additional file 9. Contribution of predictors to HAB + SPAT models. Independent contribution of different predictors to the best HAB + SPAT model. [file 12898_2018_205_MOESM9_ESM.docx]

**Additional file 9**

**Fig S9**  Independent contribution to the best habitat+spatial model explained by habitat and spatial predictors.

**
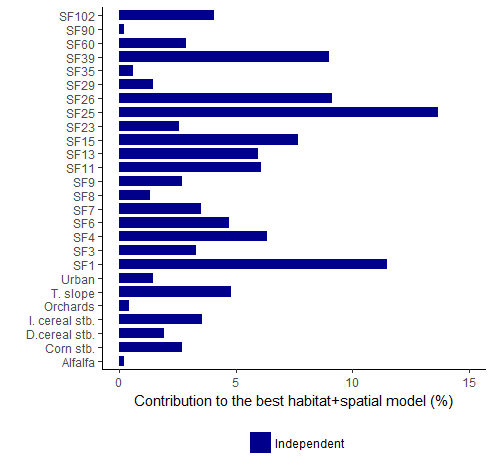
**
